# Modification of Hydrotalcite Loading Tannic Acid with Organic Silane and Application in Anticorrosive Epoxy Coating

**DOI:** 10.1002/open.202400120

**Published:** 2024-06-28

**Authors:** Bui Minh Quy, Nguyen Thuy Chinh, Nguyen Thi Kim Anh, Vu Thi Tuyet, Nguyen Xuan Thai, Vu Quoc Trung, Ngo Thi Cam Quyen, Nguyen Ngoc Tan, Thai Hoang

**Affiliations:** ^1^ Thai Nguyen University of Sciences Thai Nguyen University Tan Thinh Thai Nguyen Vietnam; ^2^ Institute for Tropical Technology Vietnam Academy of Science and Technology 18 Hoang Quoc Viet Cau Giay Ha Noi Vietnam; ^3^ Graduate University of Science and Technology Vietnam Academy of Science and Technology 18 Hoang Quoc Viet Cau Giay Ha Noi Vietnam; ^4^ Faculty of Chemistry Hanoi National University of Education 136 Xuan Thuy Cau Giay Ha Noi Vietnam; ^5^ Institute of Environmental Sciences Nguyen Tat Thanh University Ward 13 District 4 Ho Chi Minh Vietnam; ^6^ Hong Ha Shipbuilding Company Km 17+300 National Highway, Le Thien Commune, An Duong District Hai Phong Vietnam

**Keywords:** organic modification, tannic acid, hydrotalcite loading tannic acid, epoxy, corrosion resistance

## Abstract

Metal corrosion is a challenge for the world with heavy impacts on the economy. Study on the development of effectiveness anticorrosion additives is a promising strategery for the protection industry. This research focuses on the modification of hydrotalcite Mg−Al (HT) loading tannic acid (TA) with 3‐(trimethoxy silyl) propyl methacrylate organo‐silane (TMSPM) for applicating as an anti‐corrosion additive for epoxy coating on the steel substrate. The suitable ratio of HT and modifiers was investigated and the suitable content of modified HT in epoxy matrix was found based on mechanical properties of the epoxy‐based coating. The characteristics of modified HT were assessed through infrared (IR) spectroscopy, X‐ray diffraction pattern (XRD), scanning electron microscopy (SEM), thermal gravimetry analysis (TGA), water contact angle (WCA), dynamic light scattering (DLS). Detailly, HT‐TA3‐S3 shows good stability in distilled water when HT/TA was modified with TMSPM which makes Zeta potential decreases significantly. Besides, SEM analysis presented HT‐TA‐S has a cylindrical shape about of 500 nm. Moreover, the crystallite size of HT/TA after being modified by TMSPM decreases sharply. All of these prove successfully synthesize HT loading TA with modified TMSPM. Water contact angle (WCA) decreases in case of loading TA and increases in case of modifying with TMSPM (WCA changed from HT (116.3°) to HT‐TA (102.4°) and HT‐TA‐S (120.1°) which indicates the increased hydrophobicity of the sample. The obtained results showed HT/TA was modified successfully with TMSPM. The modification affected the size distribution and surface properties of HT nanoparticles while it did not impact on the crystal structure of HT. After incorporating modified HT/TA into the epoxy coating, the adhesion of coating to steel substrate was improved significantly. Consequently, the adhesion of epoxy/3 wt. % modified HT/TA coating was increased 3 times as compared to epoxy neat (from 0.76 MPa to 2.77 MPa). In addition, the relative hardness and gloss retention of epoxy/3 wt. % modified HT/TA coating reached the maximum values as compared to the others. Owing to salt spraying results, the epoxy/3 wt. % modified HT/TA exhibited an excellent anticorrosion ability for the steel substrate. All the above results show the potential of HT nanoparticles loading TA modified with TMSPM as anticorrosive additives for protective coatings on steel substrates.

## Graphical abstract

Hydrotalcite Mg‐Al (HT) loading tannic acid (TA) modified 3‐(trimethoxy silyl) propyl methacrylate organo‐silane (TMSPM) (HT‐TA‐S) has been prepared and applied as an anti‐corrosion additive for epoxy coating on the steel substrate. The epoxy coating sample contains 3 wt. % HT‐TA3‐S3 shows great mechanical properties and anticorrosion ability.

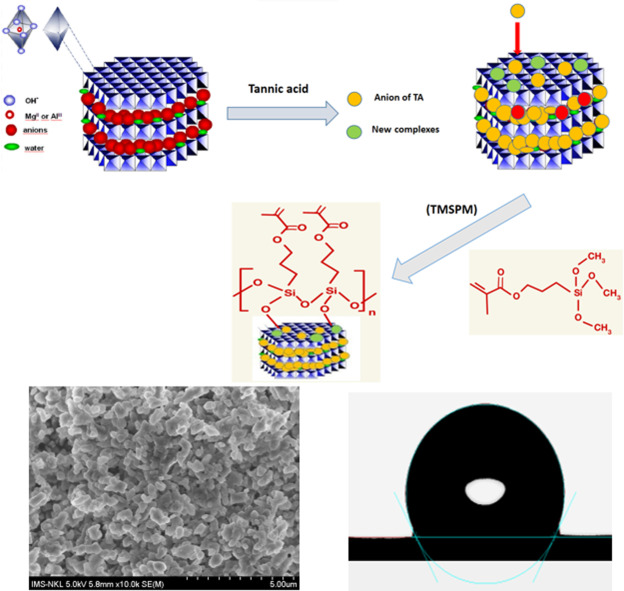

## 1 Introduction

Metals and alloys have a wide range of applications in various fields of industry and living due to their unique properties. These materials play a crucial role in modern technology and infrastructure, making them essential components in many products and structures.[^1^, ^2^, ^3^] Recently, metal corrosion has become a sore problem in the world. Metal corrosion affects many aspects of life such as health, the environment and especially seriously affects industry around the world. This has caused enormous losses to industries with the worldwide annual cost of metal corrosion around US$ 2.5 trillion.^[4]^ Therefore, metal corrosion prevention solutions are extremely important to protect and maintain metal structures and equipment in diverse and harsh environments.

Currently, there are many ways in which metal corrosion can be prevented and controlled including the use of high‐performance corrosion‐resistant metals, protective coatings on metal surfaces, electrochemical technology, oxidation and phosphorylation of metal surfaces and the addition of corrosive agents, inhibitors, additives, etc.^[5]^ Qiao *et al*. has used passivation technique of 718 alloys in acidic solutions;^[6]^ Zhang *et al*. have utilized alloying technique by adding Calcium (Ca) as an alloying element to improve the corrosion properties of the AZ61‐Nd magnesium alloy;^[7]^ resistant coatings (polymer‐based coatings) is also studied by Cai *et al*. which has the use of waterborne polyurethane (PU) coatings and their nanocomposites as protective and anticorrosive coatings for metal substrates, etc.^[8]^ Among them, the application of organic polymer coatings is considered as an easily developed measure to protect against corrosion of metal materials. Waterborne solvent epoxy coating is commonly used in anti‐corrosion paints due to their superior mechanical properties, high adhesion to the substrate surface, good chemical resistance, and low cost. However, waterborne solvent epoxy coatings have defects such as microscopic pores in their structure that were formed by the evaporation of the solvent, making the coating permeable to aggressive agents (i. e. water, oxygen, Cl^−^). All of these can lead to corroded metal surfaces increasing over time.[^9^, ^10^] Sometimes, the corrosion process can be also influenced by the used metal substrate or the treatment process.[^11^, ^12^] Gong *et al*. studied the degradation process of the epoxy‐based coating on three kinds of metal substrates (carbon steel, Al alloy, and brass) and recognized that the characteristics of the metal substrates and their corrosion products affect the coating failure behavior, the same coating on the different substrate exhibits different failure times.^[11]^ Adding inorganic nanoparticles to epoxy coatings is a promising method to enhance the corrosion protection performance of epoxy coatings because these particles can help fill microscopic pores in the structure of epoxy and produce nanocomposite coatings with long‐term corrosion resistance.^[9]^ Moreover, epoxy not only can create a three‐dimensional spatial network, helping to fix and link the components in the composite but also can melt and harden, forming a coating and bonding between the components in the composite.^[13]^ Besides, epoxy also has potential applications including aerospace, automotive, and infrastructure sectors where strong and lightweight composite materials are in demand.^[14]^ Therefore, to overcome the above disadvantages of epoxy, this research focuses on the effective application of Mg−Al hydrotalcite modified with tannic acid and organic silane in epoxy coatings.

Magnesium‐aluminum hydrotalcite (abbreviated as HT) is a magnesium‐aluminum hydroxycarbonate with the formula Mg_6_Al_2_CO_3_(OH)_16._4H_2_O.^[15]^ The layered structure of HT consists of positively charged hydroxide layers, and the interlayer consists of carbonate anions and water molecules.[^16^, ^17^] The HT is synthesized quite easily, and it is stable under normal conditions. The decarbonization temperature is about 330–370 °C.[^18^, ^19^, ^20^] There are several studies synthesizing HT or hybrid HT materials using co‐precipitation method combined with hydrothermal treatment.[^16^, ^21^, ^22^, ^23^, ^24^, ^25^] Surface modification is one technique that has been and is being used to change the surface of HT to expand its application. Surface modification can be performed by physical or chemical modification methods. Among them, the use of functionalized organic agents such as organic silanes and organic titanates is quite common to modify HT's surface. Zhu and his co‐workers used phenyltrimethoxysilane (PTMS) to modify HT Mg−Al through *in‐situ* process to change the surface properties of HT from hydrophilic to hydrophobic, and to shift anions in the layer converted to an organic mineralized form with a wider interlayer distance.^[26]^ Tao *et al*. studied on the effect of sodium dodecyl sulfate (SDS) concentration on the layered structure of Mg_6_Al_2_(OH)_16_CO_3_ ⋅ 4H_2_O (LDH) modified with 3‐aminopropyltriethoxysilane (APTS) by *in‐situ* co‐precipitation method.^[27]^ The modification with organic silanes can affect the surface properties, layer structure as well as the crystal structure of HT.[^28^, ^29^] 3‐(trimethoxysilyl)propyl methacrylate (TMSPM) consisting of a methacrylate ester group in its structure, is favorable to modify nanoparticles with many hydroxyl groups on the surface like HT. In some reports, TMSPM has been used to modify organic nanoparticles such as nanosilica,^[30]^ nanotitania,[^31^, ^32^] nanozirconia.^[33]^ The appropriate content of TMSPM for modifying the above nanoparticles is 3 wt. % compared to the mass of nanoparticles. After modification, the dispersibility of modified nanoparticles in polymer matrices, like acrylic emulsion resin or epoxy resin becomes better, leading to significant improvement in the mechanical, thermal properties, and weather resistance.^[32]^

In fact, the anticorrosion ability of modified HT has been confirmed. Layered double hydroxide Mg−Al modified with sodium tripolyphosphate (STPP) exhibited a good anti‐corrosion protective ability in epoxy coating for the steel substrate.^[34]^ Hang *et al*. used layered double hydroxide Mg−Al for loading inhibitors to improve the anticorrosion ability of epoxy coatings.[^35^, ^36^, ^37^] Epoxy resin has been widely used as protective coatings.[^38^, ^39^, ^40^, ^41^]

Tannic acid (TA) is a large molecular polyphenol complex. As an essential ingredient in rust converters or coatings, TA exhibits excellent anti‐metal corrosion properties when used at concentrations of 5–15 g/L.[^42^, ^43^, ^44^, ^45^] The molecular structure of TA contains many hydroxyl groups, which can form stable complexes with Fe^3+^ ions[^46^, ^47^, ^48^, ^49^] and control the transformation of the γ‐FeOOH crystalline phase into the Fe_3_O_4_ crystalline phase in the layer rust.[^50^, ^51^, ^52^, ^53^] Therefore, the TA‐based rust converter can inhibit electrochemical corrosion in corroded steel and improve the corrosion resistance of rusted steel. However, TA is a weak acid, the pH value has a significant influence on the properties of coatings. Therefore, finding the carriers for loading TA is a great way to limit the negative effect of acid environment of TA. The HT is such a carrier because it has very good adsorption capacity.

This study focuses on the modification of HT nanoparticles loading TA by TMSPM and application them in epoxy coatings. The content of TA in HT will be investigated while the concentration of TMSPM will be fixed at 3 wt. % as referred from previous studies.[^31^, ^32^, ^33^] The characteristics of modified HT/TA particles will be assessed before introducing the epoxy coating. The effectiveness of modified HT/TA particles in anticorrosion protection for steel substrates will be studied and discussed.

## Experimantal

### Materials

Hydrotalcite (magnesium‐aluminum hydroxycarbonate, HT, 99 %), and 3‐(trimethoxysilyl)propyl methacrylate (99 %, TMSPM) were provided by Sigma Aldrich. Tannic acid (97 %, TA) is a Belgian commercial product. Epoxy resin (Epikote™ Resin 1001‐X‐75, equivalent epoxy group of 450—500 g/eq, viscosity of 8—13 pa.s^−1^, 75 % solid content in xylene), polyamide hardener (amine index of 310±20 mg KOH/g, viscosity (at 25 °C) of 200~500 cPs) were purchased from Epochem company, China. Other chemicals such as ethanol (99.5 %), ammonia (25 %), xylene (99 %) were analytical chemicals.

### Preparation of Samples

#### Preparation of HT Loading TA and Modification it with TMSPM

The preparation of HT loading TA was presented as follows: First, an exact amount of TA (0.01 g, 0.03 g, and 0.05 g) was weighed and dissolved in 10 mL of distilled water. Next, 90 mL of ethanol 99.5 % was added into this solution, following 1 g of HT was dispersed in the solution. The mixture was then continuously stirred in a magnetic stirrer at 50 °C for 2 hours before centrifuging, filtering, and washing with distilled water three times to collect the solid part. To obtain the samples in dry powder, the solid part was dried at 80 °C for 4 hours to obtain HT/TA samples.

**Figure 1:**
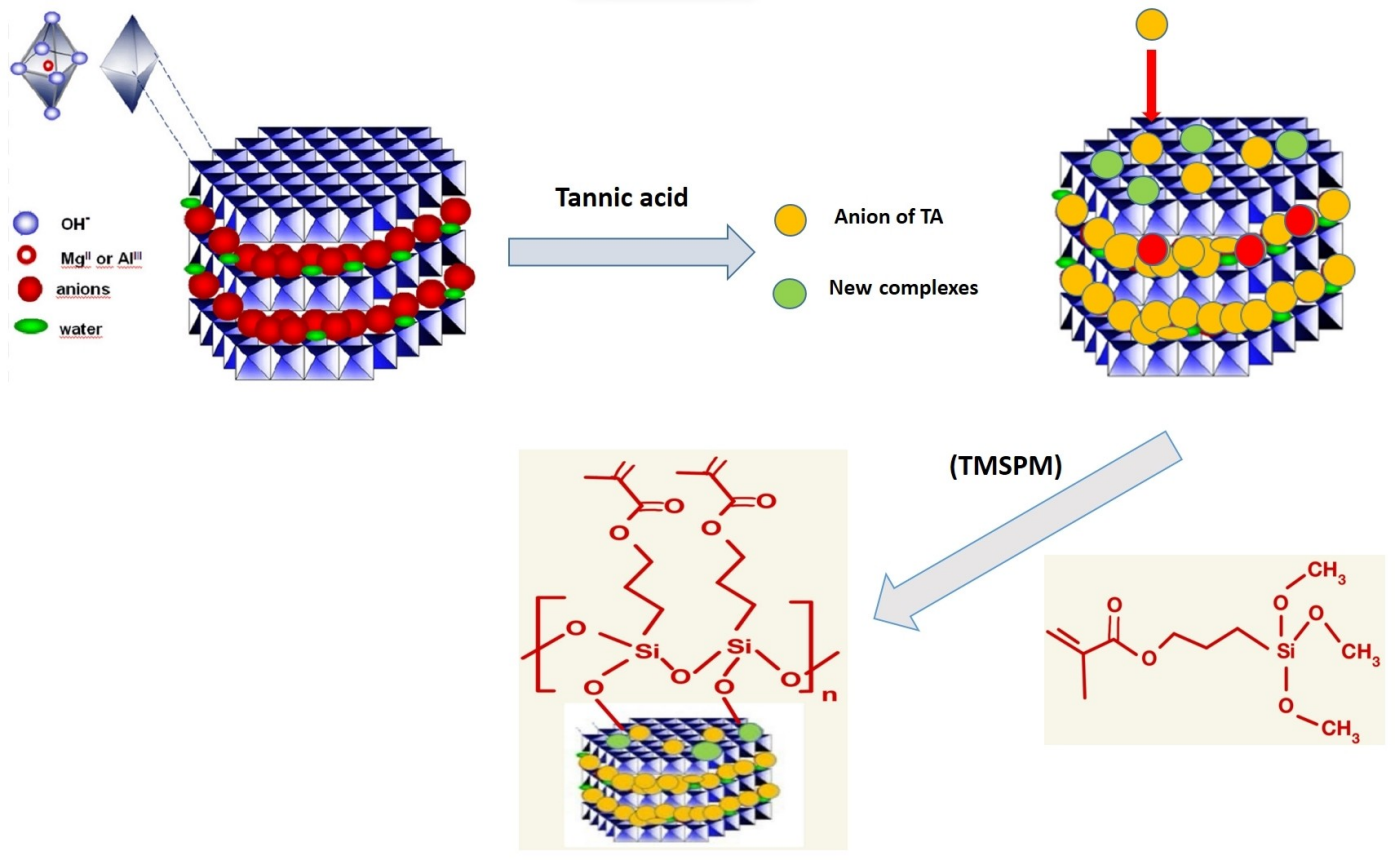
*Scheme of process of HT loading TA modified TMSPM*.^*[54]*^

A solution containing 0.03 g of TMSPM and 0.03 g of TA, 100 mL of ethanol 99.5 %/distilled water solution (90/10 v/v), and 1 mL of 25 % ammonia was prepared by stirring at 50 °C for 30 minutes to hydrolyze silane. Next, the HT was incorporated into this solution and the mixture was stirred for 3 hours at 50 °C before centrifuging, filtering, and washing with ethanol three times to collect the solid part. To obtain the samples in dry powder, the solid part was dried at 80 °C for 4 hours to obtain HT/TA modified with TMSPM (HT/TA/S). Three samples prepared with different ratios of TA/HT as 0.01/1, 0.03/1, and 0.05/1 were designated as HT‐TA1, HT‐TA3, and HT‐TA5, respectively. The HT‐TA1, HT‐TA3, and HT‐TA5 samples modified with 3 wt. % of TMSPM were abbreviated as HT‐TA1‐S3, HT‐TA3‐S3, and HT‐TA5‐S3, respectively. Figure 1 presents the scheme of process of HT loading TA modified TMSPM.

#### Preparation of Organic Coatings Based on Epoxy Resin and HT With and Without Modification

The epoxy‐based coatings were prepared using the magnetic stirrer combined with ultrasonication, in which the HT with and without modification was dispersed in 5 mL of xylene for 1 hour before incorporated in epoxy resin. Upon mixing epoxy and HT, the polyamide hardener was added into the mixture and stirred for 10 minutes before applying on the steel substrate (C45 steel, Duc Thanh Steel Co., Vietnam) using a K‐303/V film forming applicator. The wet film thickness is 120 μm, corresponding to approximate 30–35 μm when drying. Finally, the samples were allowed to dry naturally and stabilized for 72 hours for further studies. All the experiments were conducted at room temperature. The component proportions and designations of samples are given in Table 1.

**Table 1:** *Proportions of components in the coating and designation of samples*.

No.	Epoxy (g)	HT (g)	HT‐TA (g)	HT‐TA‐S (g)	Hardener (g)	Sample abbreviation
1	30	0	0	0	20	E0
2	30	0.72			20	E/3HT0
3	30		0.72*		20	E/3HT‐TA1
4	30		0.72**		20	E/3HT‐TA3
5	30		0.72***		20	E/3HT‐TA5
6	30			0.72*	20	E/3HT‐TA1‐S3
7	30			0.72**	20	E/3HT‐TA3‐S3
8	30			0.72***	20	E/3HT‐TA5‐S3
9	30			0.24**	20	E/1HT‐TA3‐S3
10	30			1.20**	20	E/5HT‐TA3‐S3

*HT‐TA1 or HT‐TA1‐S3 **HT‐TA3 or HT‐TA3‐S3 ***HT‐TA5 or HT‐TA5‐S3.

### Characterization

Infrared (IR) spectra of samples were performed using a Nicolet iS10 spectrometer (USA) in the wavenumber ranging from 4000 cm^−1^—400 cm^−1^ at room temperature with a resolution of 16 cm^−1^, and 32 scans. HT samples with and without modification were pressed into KBr pellets and recorded transmittance spectra while epoxy‐based coatings were recorded attenuated total reflectance spectra.

The morphology of nanoparticles and coatings was assessed using a field emission scanning electron microscopy (FESEM) S‐4800 device (Hitachi, Japan). The samples were coated with a thin layer of platinum to increase conductivity.

The size distribution, average particle size and Zeta potential of HT‐based nanoparticles were measured on a SZ‐100Z2 nanoparticle size and Zeta potential analyzer (Horiba, Japan). 0.01 g of samples was weighed and dispersed in 10 mL of distilled water by ultrasonication before determining the size distribution and average particle size.

Thermal characteristics of HT samples were evaluated by thermogravimetric analysis (TGA) method on a TG60H device (Setaram, Japan) in an air environment with a heating rate of 10 °C/minutes in temperature ranging from room temperature to 600 °C.

X‐ray diffraction (XRD) patterns of HT samples were recorded on a SIEMENS D5000 device (Germany) in 2 theta ranging from 2 to 80° with a CuKα irradiation (λ_max_ of 0.154 nm), a step of 0.030°, and a scanning speed of 0.043°/seconds.

The water contact angle (WCA) of the samples was determined on a Phoenix‐150 device (Korea).

The adhesion of epoxy‐based coatings on the steel substrates was evaluated on an Elcometer F510‐20T automatic coating adhesion tester (UK) according to ASTM D968‐15 standard. The relative hardness of samples was determined using an Erichsen model 506 (Germany) according to ISO 1522. The measurement was repeated 5 times, and the obtained result is mean±SD.

### Assessment of Anticorrosion Protection Ability

The salt spray test was performed on Q‐FOG equipment (USA) according to ASTM B117 standard. The coating was scratched with an X‐shaped incision according to ISO 17872. The coating samples were photographed and evaluated for rust stains according to ASTM D1654 after 168 hours of testing.

## 2 Results and Discussion

### 2.1 Characteristics and Properties of HT‐Based Nanoparticles

#### 2.1.1 Infrared Spectrum (IR)

**Figure 2:**
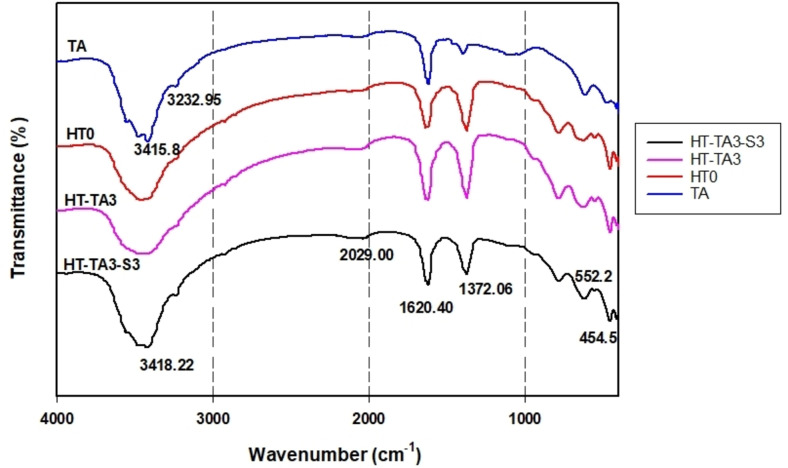
*IR spectra of tannic acid (TA), hydrotalcite (HT0), hydrotalcite loading TA (HT‐TA3) and hydrotalcite loading TA modified with TMSPM (HT‐TA3‐S3)*.

Figure 2 presents the IR spectra of TA, HT, HT‐TA3 and HT‐TA3‐S3 samples. As seen from the IR spectrum of TA, some peaks characterizing for the stretching vibrations of O−H bond participating in hydrogen bonds at the wavenumber range of 3300–3600 cm^−1^, the bending vibration of O−H bond at 1619 cm^−1^, the bending vibration of C−H bond at 1397 cm^−1^, the stretching vibration of C−O bond at 1096 cm^−1^ were observed.[^52^, ^53^] The IR spectrum of HT in Figure 2 exhibited some peaks attributed to the stretching and bending vibrations of O−H bond and water molecules in layer structure at 3457 cm^−1^ and 1634 cm^−1^, respectively.[^20^, ^26^] Besides, the vibration of CO_3_^2−^ group in layer structure at 1371 cm^−1^ and 2034 cm^−1^,^[28]^ the vibration of M‐OH bond (M=Mg, Al) at 554 cm^−1^ and 454 cm^−1^.[^28^, ^29^] In the HT/TA spectrum, it can be seen that the wave range from 3200–3600 cm^−1^ has become much wider and blunter than the peak representing the hydroxyl group of HT or TA. This can be said that anions in the double layer structure of HT can be partially replaced by anions of tannic acid.

When modifying HT loading tannic acid with TMPSM, the position of peaks characterized for O−H group shifts slightly, indicating that the modification affected the functional groups on the surface of HT through the formation of physical interactions between silanol groups and hydroxyl groups on the surface of HT/TA.[^32^, ^33^] This can lead to change the surface properties of HT/TA from hydrophilic to hydrophobic as mentioned by Zhu *et al*.^[26]^ The peaks characterizing the M‐OH bond and CO_3_^2−^ remain unchanged, however, the intensity of these peaks exhibits a small decrease, may be due to the covering of silane on the surface of the nanoparticles during modification.

In addition, Anh *et al*. studied the modification of zinc aluminum hydrotalcite with 2‐benzothiazolythio‐succinic acid (BTSA) and 2‐aminoethyl‐3‐aminopropyltrimethoxysilane (APS), the spectroscopy of HT‐BTSA‐ S show characteristic peaks of Si−O−Al, Si−O−Zn at 994 cm^−1^.^[55]^

#### 2.1.2 Size Particle and Zeta Potential

Table 2 presents the Z‐average particle size (ZP), polydisperse index (PI) and Zeta potential of HT‐based nanoparticles. From Table 2, the ZP and PI values of HT0 are the highest values among tested sample, suggesting that the HT exhibits a poor hydrodynamic durability in distilled water.^[56]^ When dispersed in water, HT0 nanoparticles agglomerate to a film and then float on the water surface. This also causes a Zeta potential value of HT close to zero.

**Table 2:** *Average particle size, particle size peak, and PI of HT samples with and without modification*.

No.	Sample	Average particle size (nm)	PI	Zeta potential (mV)
1	HT0	6369.5±500.7	7.393	−0.2±−0.004
2	HT‐TA1	681.3±24.1	0.586	−49.5±−0.99
3	HT‐TA3	1158.0±13.9	0.857	−46.3±−0.926
4	HT‐TA5	1699.2±13.9	0.868	−42.0±−0.84
5	HT‐TA1‐S3	595.3±84.4	0.661	−52.0±−1.04
6	HT‐TA3‐S3	609.1±12.18	0.706	−47.7±−0.954
7	HT‐TA5‐S3	1870.0±43.1	0.993	−36.6±−0.732

**Figure 3:**
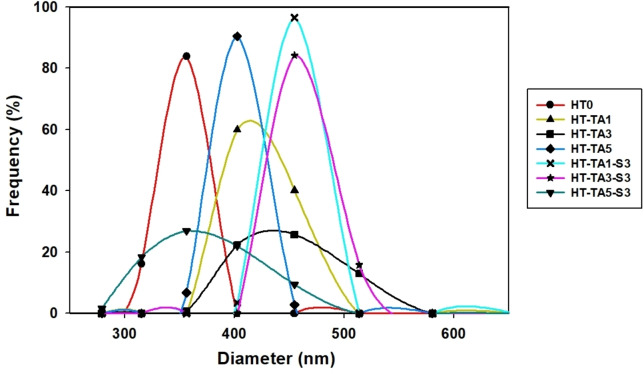
*Size distribution diagram of unmodified HT (HT0), HT loading TA (HT‐TA) and HT loading TA modified TMSPM (HT‐TA‐S)*.

From the Table 2, it is observed that after loading with TA and modification with TMSPM, the average particle size of all samples decreases significantly, especially after modification with TMSPM, ZP of HT‐TA3‐S3 decreased by a half time compared to HT‐TA3 (from 1158.0±13.9 to 609.1±12.18). This can be seen that TMSPM has a significant effect on the average size. For HT/TA and HT/TA modified TMSPM, the PI values are less than 1, showing that these nanoparticles can disperse in water thanks to the adsorption of TA on the HT.^[56]^ The ZP of their particles ranked as HT‐TA5‐S3>HT‐TA5>HT‐TA3>HT‐TA3‐S3>HT‐TA1‐S3>HT‐TA1. This exhibits that the modification led to the slight increase in ZP of HT/TA nanoparticles. When increasing TA, the increase in the size of HT/TA nanoparticles may be caused by the adsorption of TA into the layer structure of HT as well as the re‐construction of the layer structure of HT by the exchange between carbonate anions with TA anions. This can be deeply assessed through the morphological analysis as mentioned below. The ZP values of sample is larger than the particle size peak. Their results showed that HT/TA and modified HT/TA nanoparticles are highly polydisperse in water with a wide particle size distribution range.^[56]^ As viewed from Figure 3, the particle size of samples ranges from 279.04 nm to 580.41 nm. In particular, the peak sizes of the samples are respectively 356.20 nm with a frequency of 83.84 %, 402.44 nm (at frequency=59.89 %), 454.69 nm (at 25.69 %), 402.44 nm (90.43 %), 454.69 nm (96.53 %), 554.69 nm (84.19 %) and 256.2 nm (26.92 %) for samples HT0, HT‐TA1, HT‐TA3, HT‐TA5, HT‐TA1‐S3, HT‐TA3‐S3, HT‐TA5‐S3. Although they have the same diameter, the frequency of HT‐TA5 is higher than that of HT‐TA1 and that of HT‐TA1‐S3 is higher than that of HT‐TA3, this shows that modifying the HT‐TA sample with TMSPM has an effect on the peak sizes of samples. HT‐TA3 and HT‐TA5‐S3 exhibit the lower particle size peaks, suggesting that they have a greater hydrodynamic durability.

Zeta potential analysis indicates the stability of the distributed system. Therefore, substances with high Zeta potential values (both negative and positive values) are substances with good stability, while substances with low Zeta potentials tend to agglomerate with each other, being less stable.^[57]^ From Table 2, it can be seen that HT/TA and HT/TA/S samples have good stability in distilled water. This result is consistent with the particle size distribution results. The HT particles agglomerate and disperse poorly in water. When increasing the TA content for HT modification, a decrease in Zeta potential values indicates a decrease in particle stability, corresponding to an increase in the agglomeration of particles (larger average particle size) as shown above. When increasing the TA content during the HT modification process likely changes the surface properties of the particles. Because TA is a polar compound that can affect the charge density and hydrophilicity of particle surfaces. It can reduce the overall surface charge which leads to the decrease in Zeta potential. When Zeta potential decreases, the repulsive forces become weaker, allowing the particles to agglomerate. The modified HT samples at small TA concentrations with Zeta potential shifted to a more negative region showed increased particle stability in distilled water. In addition, Tian *et al*. studied on HT modification with 3‐aminopropyltriethoxysilane (APTES) with a change in solution pH value from 3–10, the zeta potential value of the samples changed from a positive value (about 15 mV) to negative (about −10 mV),^[58]^ this proves that the HT has been successfully modified by APTES. However, the Zeta potential of HT loading TA modified TMSPM was more negative than that of the HT modified with APTES, which shows that HT‐TA‐TMSPM is more stable. Because TA is a polar compound, it can make HT‐TA‐TMSPM have a higher surface charge than APTES–HT which makes the more negative Zeta potential. Besides, the more negative value of Zeta potential, the stabler particles. As a result, the HT loading TA modified TMSPM exhibits better stability in comparison to HT modified with APTES.

#### 2.1.3 Structural Morphology

**Figure 4:**
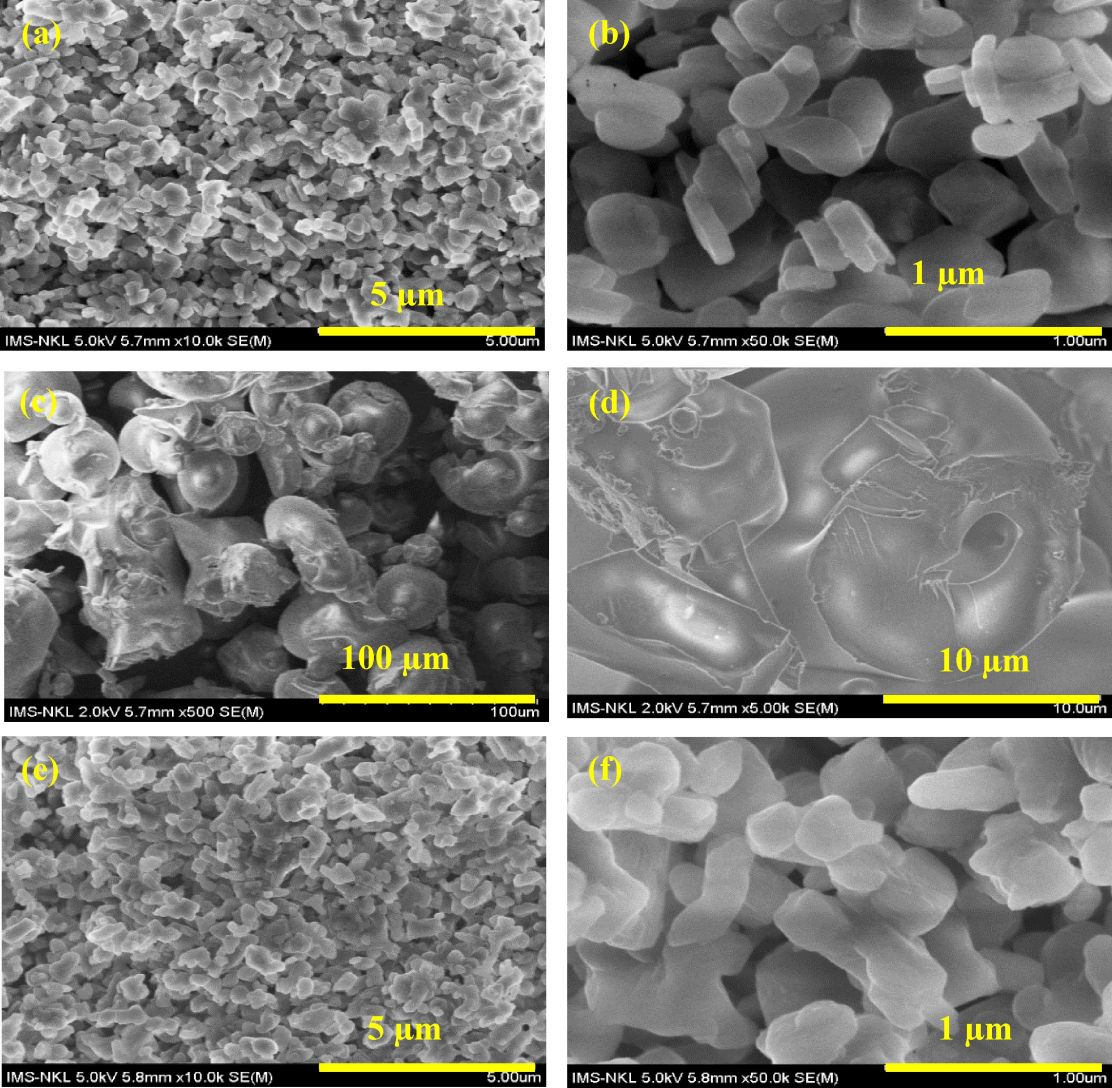
*SEM images of unmodified HT and modified HT samples at different magnifications, HT0 (a, b); HT‐TA3 (c, d); and HT‐TA3‐S3 (e, f)*.

From Figures 4a and 4b, it can be seen that the unmodified HT sample has a stacked plate structure, each with a diameter of about 500 nm and a thickness of about 400 nm. However, from Figure 4c and 4d, it is easy to see that when loading HT by TA, the shape of the HT sample is completely transformed into particles of size 20–50 mm (this may be because some anions of TA altered CO_3_^2−^ groups in the interlayer structure of HT, resulting in disruption of the layered structure of HT and causing TA loaded HT particles to clump together to form these small particles). In addition, although loading HT by TA and modification HT/TA by TMSPM also alters the shape of the original HT, it nevertheless produces TMSPM modified HT/TA particles with a cylindrical shape of about 500 nm (Figures 3e and 3 f). Moreover, in the study of modifying HT with APTES, it was shown that the thickness and the diameter of APTES modified HT (A‐HT) ranges from 32–42 nm and 110–220 nm, besides the size of A‐HT is also close to equal to the original HT, so APTES modification does not change the characteristic morphology of HT particles.^[58]^

#### 2.1.4 X‐Ray Diffraction

**Figure 5:**
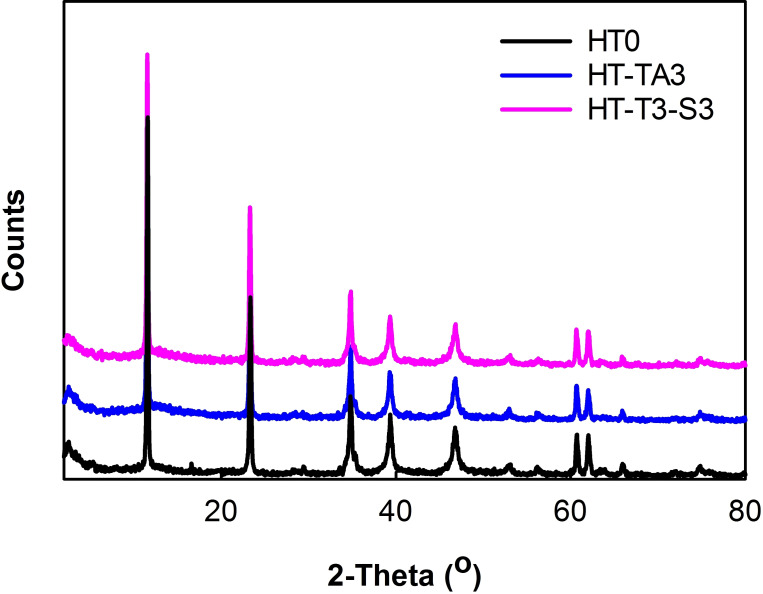
*XRD patterns of unmodified HT, HT‐TA3, HT‐TA3‐S3*.

Figure 5 is an X‐ray diffraction diagram of original HT, HT loading TA and HT loading TA modified TMSPM. Observing Figure 5, it can be seen that the diffraction peaks characterize the crystal phase structure of HT. Specifically, the strong peak at about 2 theta=11.5° corresponds to the plane (003), peak at 2 theta=23.5° corresponds to the plane (006) of HT particles interlayer structure CO_3_^2−^.[^26^, ^59^] The crystallite sizes of unmodified HT, HT loading TA and HT loading TA modified TMSPM samples are 16.393, 19.973, 7.039, respectively. It can be seen that the HT crystallite size was increased after loading TA, this may be because some anions of TA have replaced CO3^2−^ in the structural interlayers of HT, this may have affected the aggregation by HT. However, after further modification of TMSPM, the crystallite size of HT/TA was decreased sharply, which showed that silane wielded influence on the aggregation ability of HT. From Figure 5, it is not only confirming the success loading HT by TA and modification HT/TA by TMSPM, but also show that after loading with TA and modification HT/TA with TMSPM, the d‐spacings of pre‐ and post‐modification HT samples were changed not much. However, the diffraction peak intensity characterizing the planes (003) and (006) had a slight decrease indicating that the crystal structure of the post‐modification HT particles remained almost unchanged. When applying APTES for modification HT, it is noted that the crystalline structure of HT nanoparticles remains unchanged.^[58]^ This suggests that the use of silane for HT modification did not influence the original crystalline structure.

#### 2.1.5 Water Contact Angle

**Figure 6:**
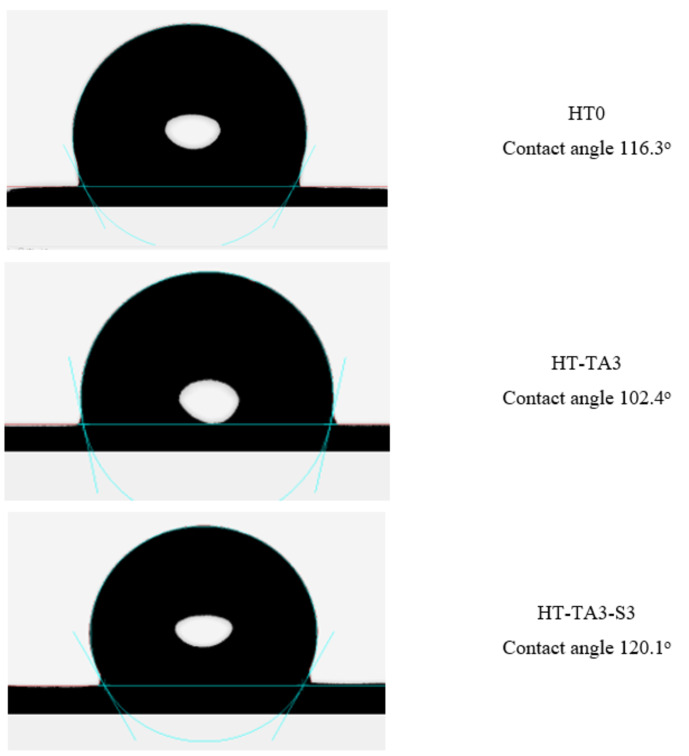
*Contact angle of original HT, HT loading TA and HT loading TA modified TMSPM*.

Figure 6 displays dynamic water contact angle images of original HT (HT0), HT loading TA (HT‐TA3) and HT loading TA modified TMSPM (HT‐TA3‐S3) samples. The sample HT0 has a hydrophobic surface with a water contact angle of 116.3°.^[57]^ After loading with TA, the water contact angle of the sample was decreased (102.4°), indicating that the surface of sample was changed from hydrophobic to hydrophilic. This is because when loading HT with TA, the TA forms polar functional groups on the surface of HT through chemical reactions. That helps to form a surface adsorption phenomenon, which can create a thin layer of water on the surface, reducing the contact angle of the HT surface with water.[^60^, ^61^, ^62^] However, after loading with TA and modification with TMSPM, the water contact angle of the sample increased (120.1°), possibly due to the grafting of organic silane (with hydrophobic organic part) onto the surface of HT, leading to increase hydrophobicity of sample. Besides, Tian *et al*. also showed that HT‐modified samples with APTES will have a gradually increasing water contact angle because the nanoparticles have high specific area, leading to an increase in surface free energy, making surface atoms more active, increasing affinity for water. This caused the surface of HT to become more hydrophobic and resulted in the contact angle gradually increasing.^[58]^

#### 2.1.6 Thermal Properties

**Figure 7:**
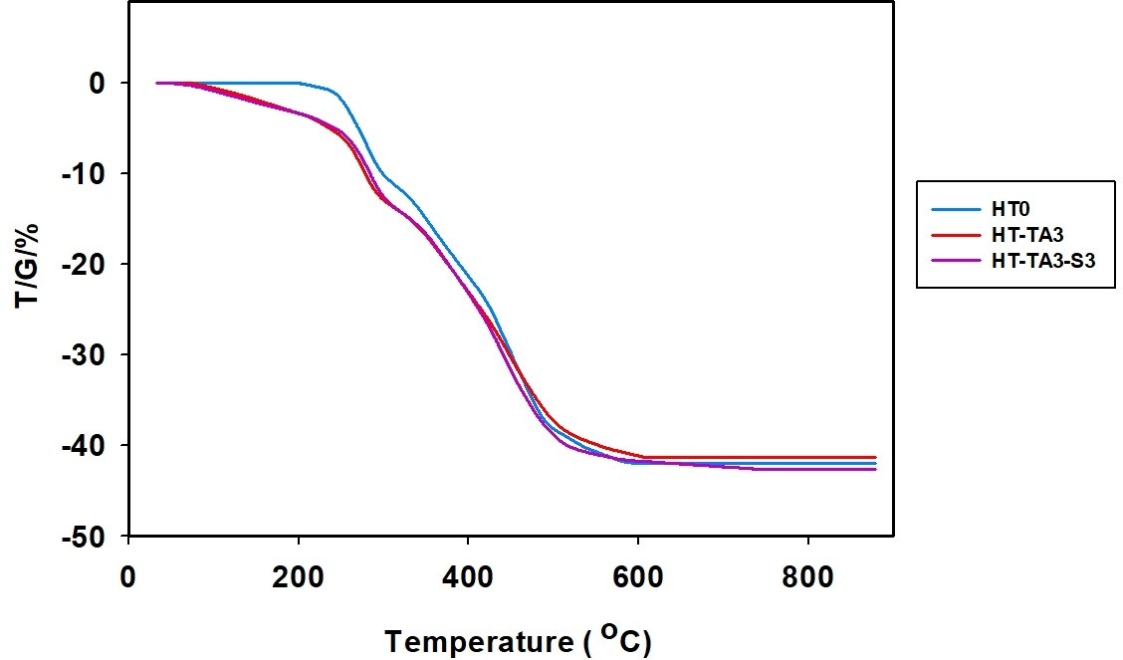
*TG diagrams of samples of unmodified HT (HT0), TA‐modified HT (HT‐TA3) and TA+TMSPM modified HT (HT‐TA3‐S3)*.

**Figure 8:**
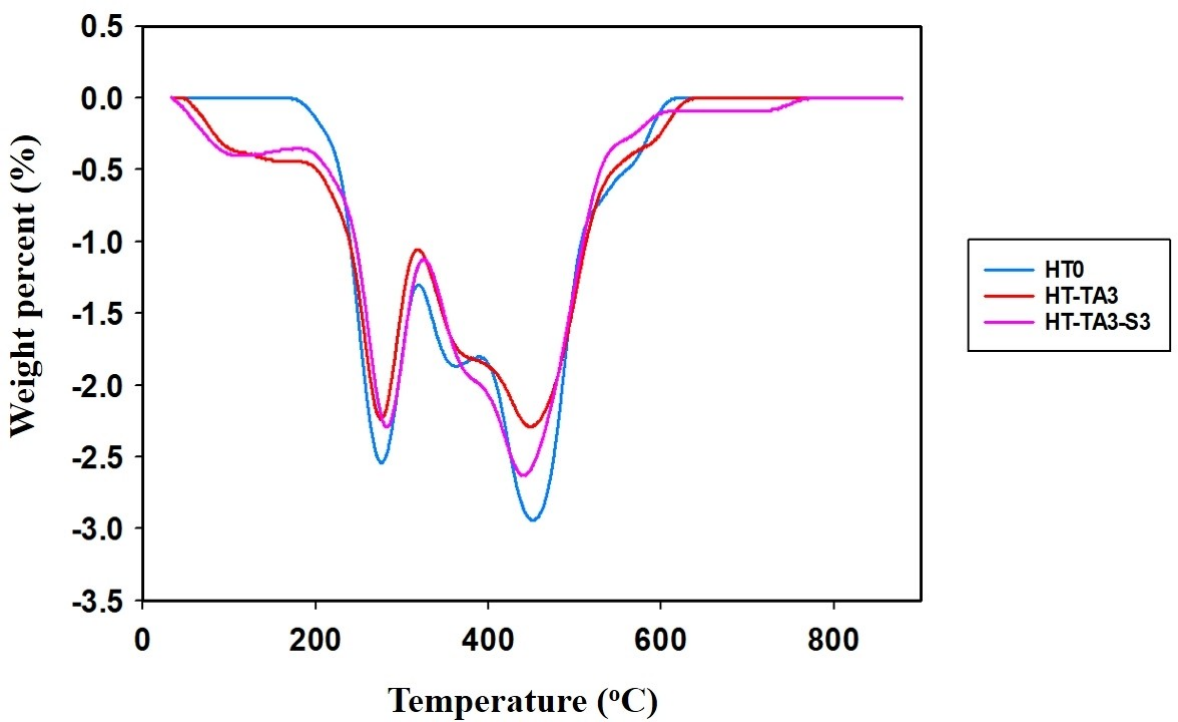
*DrTGA diagrams of samples of unmodified HT (HT0), TA‐modified HT (HT‐TA3) and TA+TMSPM modified HT (HT‐TA3‐S3)*.

Figures 7–8 are TGA diagrams of HT samples with loading and modification. It can be seen that the HT sample loses mass in 2 steps corresponding to 2 maximum decomposition temperature values (T_max_) on the differential thermogravimetric analysis (DrTGA) diagram. Observing the TG diagram of HT sample, the first step of HT decomposition is in the temperature range from 200 °C to 300 °C (T_max1_=275 °C) (degradation of 11.77 %), corresponding to the dehydroxylation of HT. In the second step, HT continues to decompose in the temperature range from 300 °C–600 °C (T_max2_=452 °C) (degradation of 30.16 %), corresponding to the decomposition of CO_3_^2−^ anions interspersed in the structure of HT to CO_2_.[^16^, ^26^] At the same time, the destruction of the layered structure of HT occurs and the formation of mixed oxides of Mg/Al, therefore, the remaining mass at 600 °C corresponding to the oxides of Mg/Al is 58.07 %.

Figures 7 and 8 are the TG and DrTGA diagrams of the HT loading TA and the HT/TA modifying TMSPM when compared with the original HT sample. The sample HT‐TA3 also decomposes in 2 steps similar to the HT sample. However, in the first step, the sample mass loss was higher than that of the HT, showing that besides the dehydroxylation of HT, there was also evaporation of water and decomposition of tannic acid in sample HT‐TA3.^[26]^ In the second step, the mass of sample HT‐TA3 was reduced lower than that of the sample HT possibly because anion of TA reacted and partially replaced CO_3_^2−^ in the interlayer space of HT, causing the decomposition of CO_3_^2−^ into CO_2_ in sample HT‐TA3 slightly was decreased (Figure 8). The remaining mass of the sample corresponding to metal oxides of Mg/Al at 600 °C was 58.66 %.

The thermal decomposition of sample HT‐TA3‐S3 also follows the same two steps as samples HT and HT‐TA3 (Figure 7 and Figure 8). However, in the first step, sample mass loss started to occur from about 100 °C and the sample had higher mass loss compared to HT and HT‐TA3 (14.58 % loss) indicating that in addition to dehydroxylation decomposition of HT also occurs with loss of water absorbed on the sample surface and decomposition of TMSPM grafted onto the surface of HT. From Figure 1, it can be observed that when loading HT samples bearing TA and modifying with TMSPM, silane is often easily hydrolyzed in water and creates silanol radicals (Si−O−M, M=Al, Mg).^[63]^ The remaining mass of the sample corresponding to metal oxides of Mg/Al at 600 °C is 57.33 %.

### 2.2 Characteristics and Properties of Epoxy/Modified Hydrotalcite Coatings

#### 2.2.1 Adhesion

**Figure 9:**
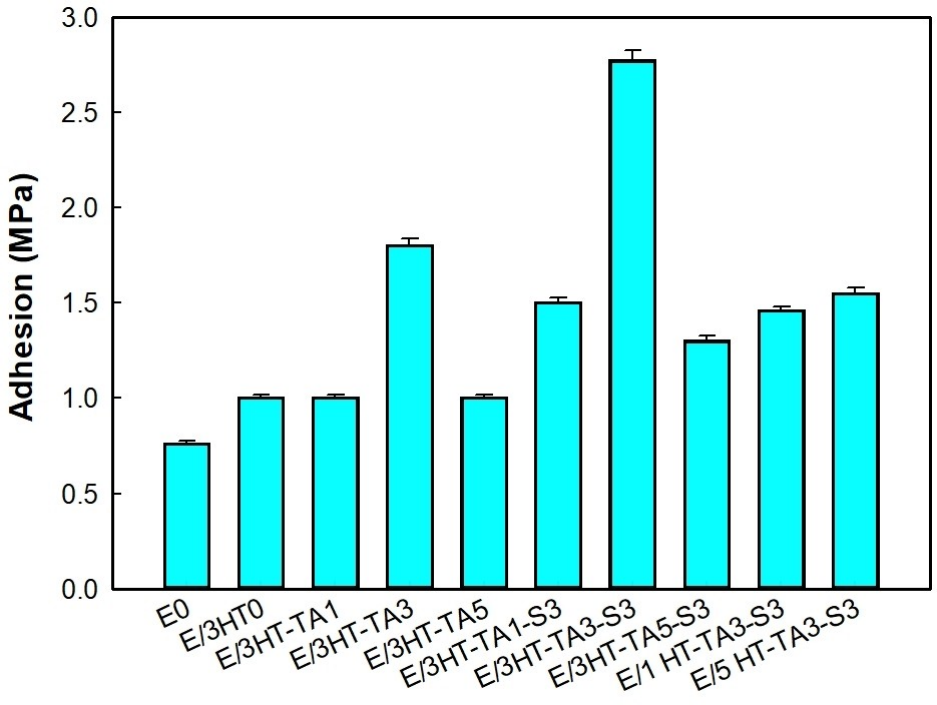
*Adhesion of epoxy‐based coatings contained original HT, HT loading TA and HT loading TA modified TMSPM*.

From Figure 9, it is easy to observe that the value of adhesion of epoxy/HT0 increases negligibly as compared to the neat epoxy. The epoxy coating contained 3 wt. % of HT‐TA3 has better adhesion, it is about 2 times more than the neat epoxy. This may be because TA also has a lot of hydroxyl groups which can form hydrogen bonds or chemical interactions with the surface of metal and epoxy. Interestingly, the epoxy containing HT modified with TA and TMSPM has an extremely increase, more than 3 times, as compared to the neat epoxy. It can be explained by silane having several functional groups such as silanol groups which can generate stronger bonds with HT/TA and coating epoxy. As a result, the 3HT‐TA3‐S3 can significantly improve the adhesion of the coating epoxy to the steel matrix. The increase in the adhesion of HT‐TA3‐S3 is also caused by the increase in hydrophilic properties of the epoxy‐based coating as discussed below. As changing the content of HT‐TA3‐S3, the adhesion of coatings was modified and reached the maximum value at the 3 wt. % HT‐TA3‐S3. The significant improvement was also observed for the resin modified with organic silane.^[64]^ The Turkey HSD test indicates that the adhesion of E/3HT‐TA3‐S3 is significantly different as compared to others. This may be due to the difference in dispersion of the HT‐TA3‐S3 particles in the epoxy matrix as shown in the below SEM analysis.

#### 2.2.2 Relative Hardness

**Figure 10:**
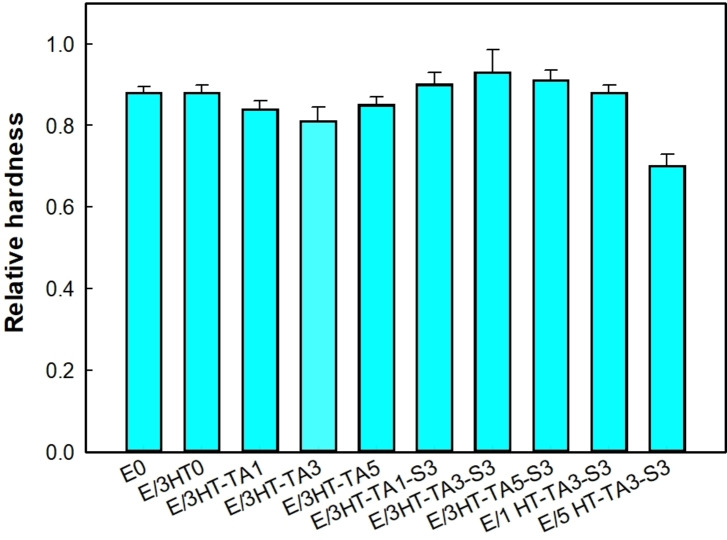
*Relative hardness of epoxy‐based coatings contained original HT, HT loading TA and HT loading TA modified TMSPM*.

Figure 10 indicates that both HT‐TA and HT‐TA−S influence slightly on the relative hardness of epoxy coating. The relative hardness of epoxy‐based coatings reaches from 0.8 to 0.95, and the relative hardness of E/3HT‐TA3‐S3 sample is highest and significantly different among tested samples. The slight improvement in relative hardness of the epoxy coating in the presence of HT‐TA3‐S3 may be due to the TMSPM modified HT/TA interacting and dispersing well in the epoxy matrix, the TMSPM organic coupling agent acts as a bridge between the epoxy resin and HT particles.

#### 2.2.3 Water Contact Angle

**Figure 11:**
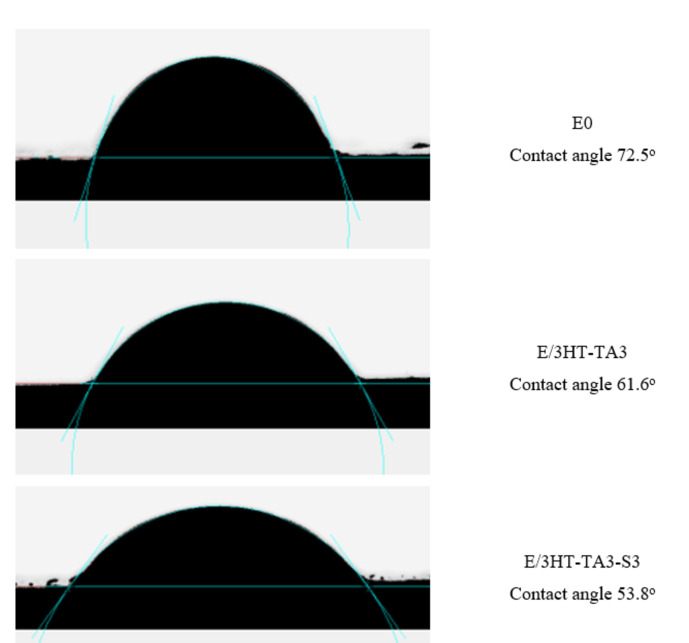
*Contact angle of epoxy‐based coatings contained HT loading TA and HT loading TA modified TMSPM*.

Figure 11 demonstrates the water contact angles of epoxy‐based coatings. The epoxy, E/3HT‐TA3, and E/3HT‐TA3‐S3 coatings have a water contact angle of 72.5°, 61.6°, and 53.8°, respectively. This result indicates that the surface of epoxy coatings becomes more hydrophilic when added modified HT particles. The reduction in water contact angle of coatings may be due to the increase in number of polar groups in the epoxy‐based coatings.^[60]^ The increase in hydrophilic of the coating enhances the ability to interact between the coating and steel substrate, thereby, increasing the adhesion of coating and steel substrate.^[65]^ This explains the significant improvement in adhesion of E/3HT‐TA3‐S3 as discussed above.

#### 2.2.4 Infrared Spectra (IR)

**Figure 12:**
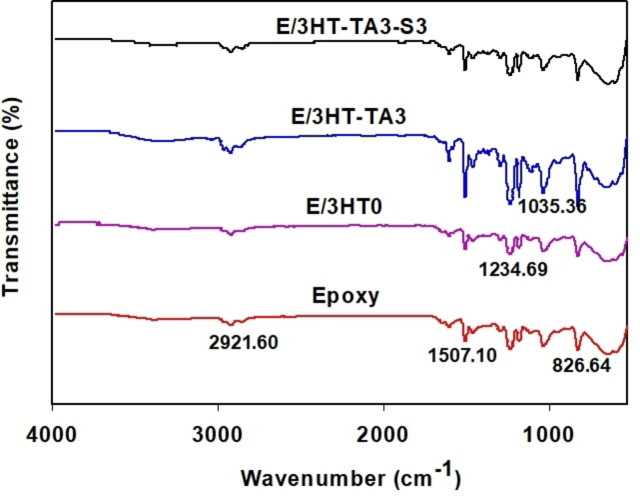
*IR spectra of epoxy‐based coatings contained original HT, HT loading TA and HT loading TA modified TMSPM*.

The IR spectra of epoxy‐based coatings are presented in Figure 12. As viewed from IR spectrum of epoxy, the vibrations of epoxy ring (at 1235 cm^−1^, 1180 cm^−1^, 1035 cm^−1^), C−H bond (at 2921 cm^−1^) and C−N bond of polyamide hardener (at 1505 cm^−1^).^[66]^ When HT is introduced to the epoxy resin, new peaks characterized for O−H bond (at 3390 cm^−1^ and 1650 cm^−1^) and CO_3_^2−^ (at 1360 cm^−1^) in HT was observed.^[67]^ For the E/HT‐TA3‐S3 sample, it can be seen the new vibrations of C=O bond (at 1720 cm^−1^), C=C bond (at 1580 cm^−1^) in TMSPM and TA of HT‐TA3‐S3. The peaks assigned for vibrations of the C−H, C=C, C−O bonds shift slightly as compared to the IR spectrum of E/3HT‐TA3‐S3 with others, suggesting that the HT‐TA3‐S3 can interact with the functional groups in epoxy resin through dipole‐dipole interactions and hydrogen bonds.

### 2.3 Anticorrosion Protection Ability of Epoxy‐Based Coatings

**Figure 13:**
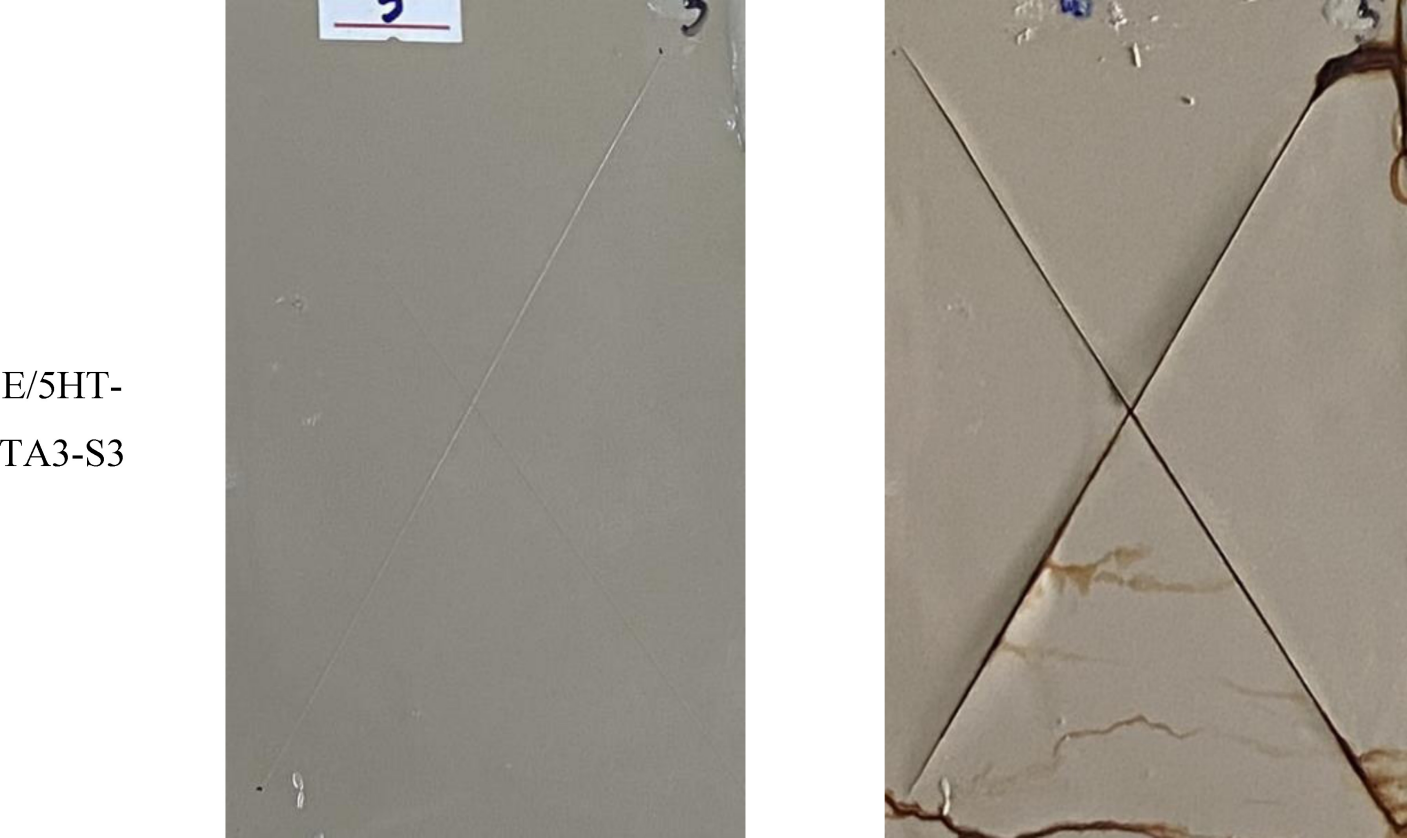
*Images of epoxy‐based coatings contained original HT, HT loading TA and HT loading TA modified TMSPM before and after 168 hours of salt spray test. The scribe was located centrally on the plate to meet the minimum distance of 20 mm to the edge (ISO 17872)*.


Figure 13 displays images of the epoxy‐based coatings before and after 168 hours of salt spray test. It can be seen that all samples are rusty at the incision line, the rust does not expand to the area outside the incision line. This can be explained by moisture, steam, and Cl^−^ ions attacking the polymer at the incision line. It causes a reaction that breaks the bonds of the polymer in the coating as well as diffuses corrosion inhibitor agents out of the coating, reducing the effectiveness of epoxy coating in protecting the steel substrate. The Cl^−^ ions attack steel surface through incisions, causing the steel substrate to corrode and produce red‐brown rust.^[53]^ At different HT‐TA3‐S3 contents, the steel substrates covered by the coating were corroded. However, in the epoxy coating sample contained 5 wt. % HT‐TA3‐S3, it was observed that the coating was blistered and peeled off the steel substrate. This may be because at a content of 5 wt. % HT‐TA3‐S3, these particles were aggregated together, creating defects in epoxy coating's structure, leading to the decrease in protection ability for the steel substrate by this epoxy coating.

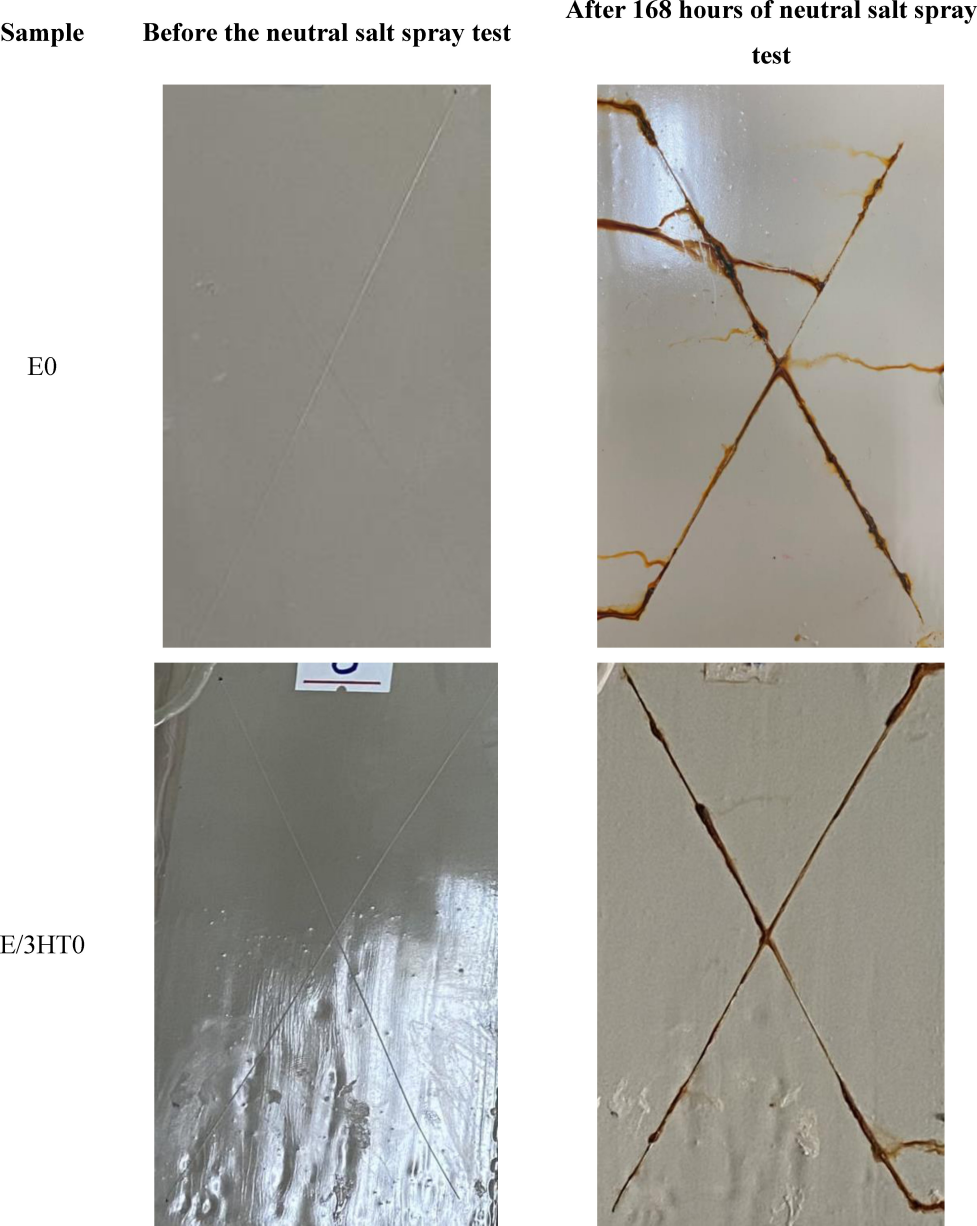



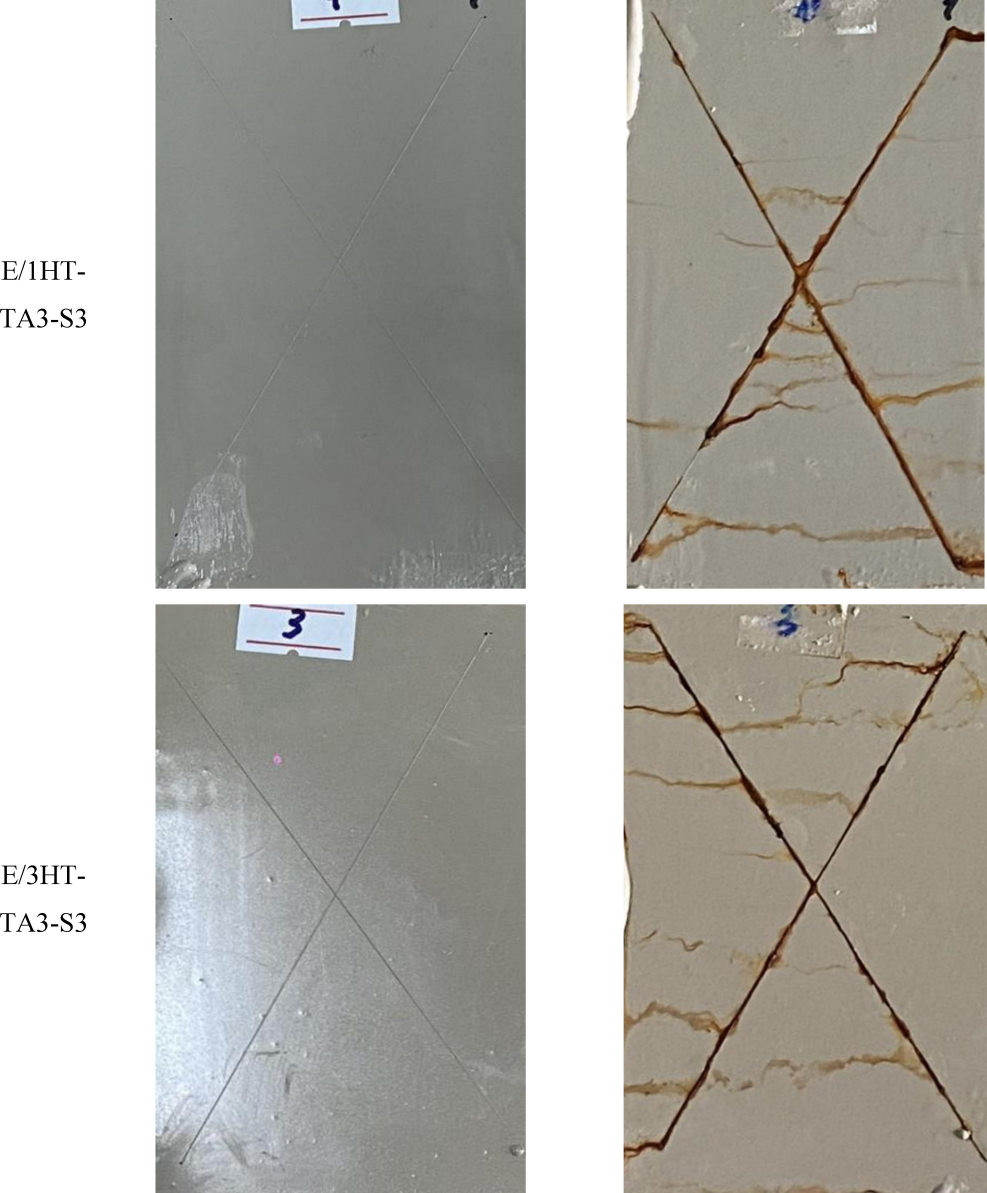


The rust stain area of the test coating samples is taken as an average of 10 measurements at points of the X‐shape on investigated samples. The rust spot area values of the epoxy‐based coatings are shown in Table 3. It can be seen that the rust spot width of the coating samples after 168 hours of salt spray test has a difference from each other. Sample E/3HT‐TA3‐S3 has the lowest rust width (0.831±0.012 mm) which shows that this sample has better corrosion resistance than the other samples. The corrosion resistance of these samples reaches level 8 according to ASTM D1654‐08 standard.

**Table 3:** *Width of rust stain of epoxy‐based coatings contained original HT, HT loading TA and HT loading TA modified TMSPM after 168 hours of salt spray test*.

Sample	Width of rust stain (mm)	Corrosion resistance according to ASTM D1654‐08
Epoxy	0.852±0.002	8
E/3HT0	0.889±0.016	8
E/1HT‐TA3‐S3	0.875±0.008	8
E/3HT‐TA3‐S3	0.831±0.012	8
E/5HT‐TA3‐S3	Unidentifiable (sample peeled off the steel base)	–

For more accurately evaluate the anti‐corrosion protection ability of epoxy coatings contained modified HT/TA particles at different concentrations, the adhesion of coating after 168 hours of salt spray test has been determined and presented in Table 4. The adhesion of all tested coatings was decreased significantly after 168 hours of salt spray test. The epoxy contained HT‐TA3‐S3 had a lower decline percentage than the epoxy coating contained unmodified and unloaded HT. This contributes to the metal anticorrosion protection ability of E/HT‐TA3‐S3 coatings.

**Table 4:** Adhesion of epoxy‐based coatings contained original HT and HT loading TA modified TMSPM after 168 hours of salt spray test

Sample	Adhesion (MPa)	% Decline
E/3HT0	0.60	40.00
E/1HT‐TA3‐S3	1.10	24.66
E/3HT‐TA3‐S3	1.97	28.88
E/5HT‐TA3‐S3	Unidentifiable (sample peeled off the steel base)	–

### 2.4 Structural Morphology

**Figure 14:**
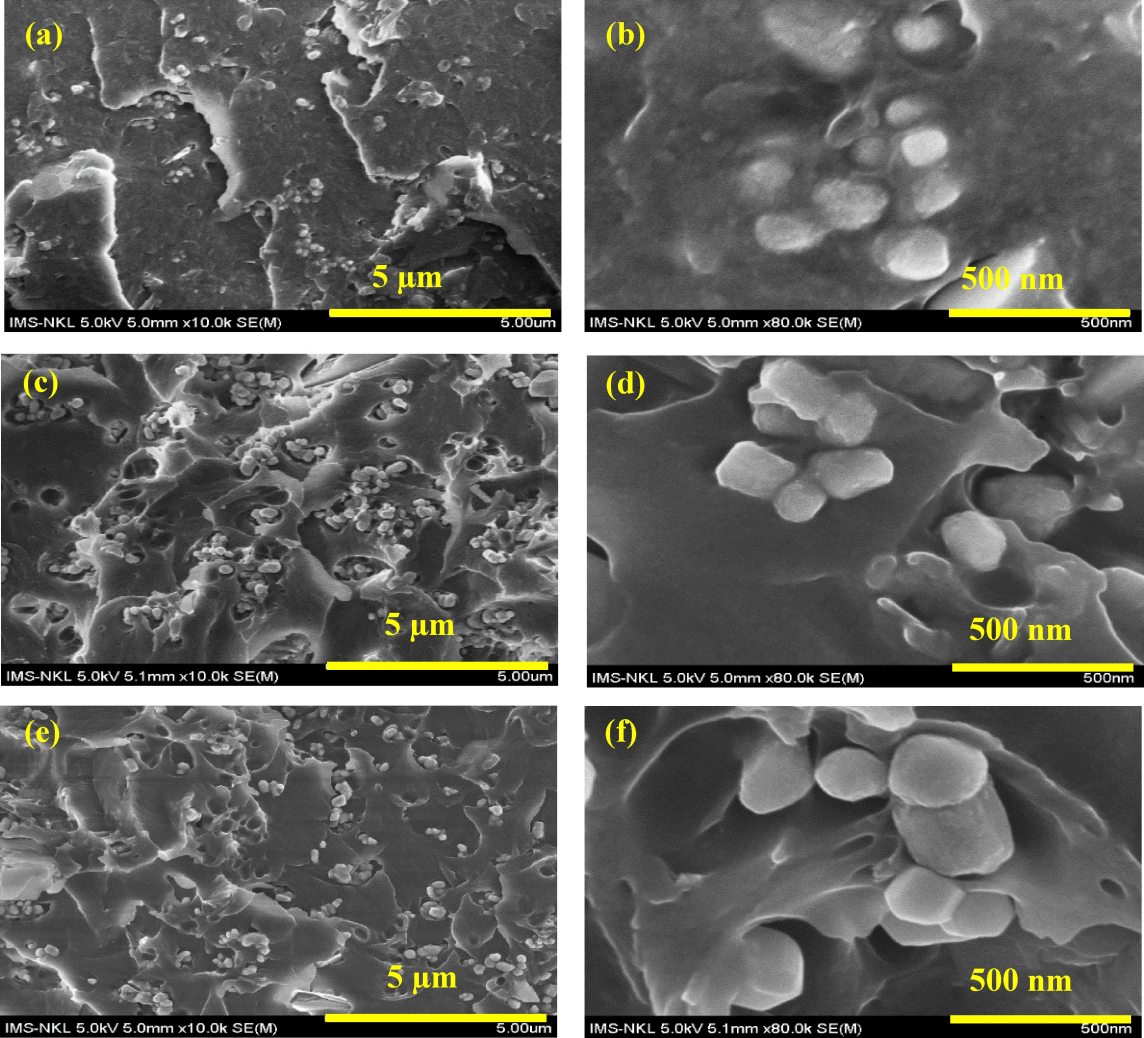
SEM image of fracture surface of E/3HT0 (a, b), E/3HT‐TA3 (c, d) and E/3HT‐TA3‐S3 (e, f) coatings

Figures 14a‐14f are SEM images of the fracture surface of epoxy‐based coatings contained *original HT, HT loading TA and HT loading TA modified TMSPM* at different magnifications (the samples were broken in a liquid nitrogen). As observed in Figures 14a and 14b, the unmodified HT clumps together when dispersed in the epoxy matrix. The basic particle size of HT particles in epoxy matrix is about 100–200 nm while the agglomerated particle size is about 500 nm–1 μm.

Observing the SEM image of the fracture surface of E/3HT‐TA3 coating in Figures 14c‐14d, the HT‐TA3 particles agglomerate together in the epoxy coating. The basic particle size of HT‐TA3 particles is about 100–300 nm while the agglomerate size in epoxy coating is about 2 μm. For E/3HT‐TA3‐S3, HT‐TA3‐S3 particles, they also tend to agglomerate but the cluster size is only about 500 nm. At the same time, when comparing to the SEM images at 10,000 times of magnification (Figures 14a, c, e), it can be seen that the HT‐TA3‐S3 particles adhere and blend to the epoxy resin better than the HT0 and HT‐TA3. This can be explained by the smaller agglomeration size in microstructure of the E/HT‐TA3‐S3 leading to reduce deformation of the coating under the impact of external stress during broking in liquid nitrogen. This helps explain the improvement in mechanical properties and metal anticorrosion protection of the epoxy coating contained HT‐TA3‐S3 particles as discussed above.

## 3 Conclusions

In this study, hydrotalcite (HT) loading tannic acid (TA) were successfully modified with 3‐(trimethoxysilyl) propyl methacrylate (TMSPM). The loading with TA and modification with TMSPM has affected the surface properties, size distribution, crystallite size and morphology of the HT. The thermal analysis results also prove the HT loading TA modified TMSPM exhibited a positive effect on the mechanical properties of epoxy coating. The increase significant in terms of adhesion shows that the strong interaction between the epoxy coating with steel substrate. Besides, the changing content of HT and TA influences the value of relative hardness. Moreover, the decrease in the water contact angle of epoxy/HT‐TA‐S3 coating also indicates the surface of epoxy‐based coating becomes more hydrophilic, that helps to adhesion with the steel substrate more easily. The epoxy/3HT‐TA3‐S3 coating exhibits a good corrosion resistance for the steel substrate. This shows the potential of applying epoxy coating contained hydrotalcite loading tannic acid modified TMSPM as a metal anticorrosion protection coating for steel structures working in marine environments.

## Author Contributions

Bui Minh Quy: Investigation, Writing‐original draft preparation. Nguyen Thuy Chinh: formal analysis, Writing‐Reviewing and Editing, Supervision. Nguyen Thi Kim Anh, Nguyen Xuan Thai: Methodology, Investigation. Nguyen Ngoc Tan, Vu Thi Tuyet: Investigation. Vu Quoc Trung, Ngo Thi Cam Quyen: Validation. Thai Hoang: Conceptualization, Funding acquisition, Writing‐Reviewing and Editing, Project administration.

## Conflict of Interests

The authors declare no competing interests.

4

## Data Availability

The data used to support the findings of this study are included within the article.
